# blast2galaxy: a CLI and Python API for BLAST+ and DIAMOND searches on Galaxy servers

**DOI:** 10.1093/bioadv/vbae185

**Published:** 2024-11-22

**Authors:** Patrick König, Anne Fiebig, Thomas Münch, Björn Grüning, Uwe Scholz

**Affiliations:** Department of Breeding Research, Leibniz Institute of Plant Genetics and Crop Plant Research (IPK), Seeland, 06466, Germany; Department of Breeding Research, Leibniz Institute of Plant Genetics and Crop Plant Research (IPK), Seeland, 06466, Germany; Department of Breeding Research, Leibniz Institute of Plant Genetics and Crop Plant Research (IPK), Seeland, 06466, Germany; Bioinformatics Group, University of Freiburg, Freiburg im Breisgau, 79098, Germany; Department of Breeding Research, Leibniz Institute of Plant Genetics and Crop Plant Research (IPK), Seeland, 06466, Germany

## Abstract

**Motivation:**

The Galaxy workflow system is an open-source platform supporting data-intensive research in life sciences, featuring a user-friendly web interface for complex analyses without extensive programming. It also offers a representational state transfer based API, enabling remote execution of specific tools. Galaxy supports similarity searches for nucleotide and amino acid sequences, with integrated tools like NCBI BLAST+ and DIAMOND. However, no specialized software currently exists for convenient use of NCBI BLAST+ and DIAMOND via the Galaxy API.

**Results:**

blast2galaxy is a Python package that uses the Galaxy API to run sequence alignments with NCBI BLAST+ and DIAMOND as Galaxy-wrapped tools on compatible servers. It includes a command-line interface that mirrors the CLI of BLAST+ and DIAMOND and a high-level Python API for direct alignments from Python applications. The package relies on bioblend for communication with the Galaxy API.

**Availability and implementation:**

blast2galaxy is available as open-source software under the MIT license. The source code is available on Github: https://github.com/IPK-BIT/blast2galaxy. It can be installed from the Python Package Index using “pip install blast2galaxy” or from the Bioconda channel using “conda install -c bioconda blast2galaxy”. Docker and Apptainer images are available and referenced in the documentation which is available under https://blast2galaxy.readthedocs.io.

## 1 Introduction

Galaxy is a web-based computational workflow execution platform widely used in biology and bioinformatics with an intuitive graphical user interface, making it accessible to life scientists who may not have extensive programming skills (The Galaxy Community *et al.* 2024). This user-friendly approach lowers the barrier to entry, allowing researchers to focus on their scientific questions rather than technical challenges.

Due to the constantly decreasing sequencing costs, more and more sequencing projects can be realized. This results in large databases of DNA and amino acid sequences. An every-day use case is that biologists want to search for specific sequences of favorite genes in a sequence database of interest. Many databases for sequence similarity search are based on the NCBI BLAST+ tools (Altschul *et al.* 1990, Altschul *et al.* 1997). Although first developed 30 years ago, the BLAST tool suite is still very popular among bioinformaticians today and is considered the gold standard for searching sequence databases with regard to sensitivity (Ward and Moreno-Hagelsieb 2014, Hernández-Salmerón and Moreno-Hagelsieb 2020). Another popular sequence alignment tool for database-based searches specialized in protein sequences is DIAMOND (Buchfink *et al.* 2021).

Galaxy can be used to host NCBI BLAST+ tools and DIAMOND, with corresponding databases of sequences for private and public usage (Camacho *et al.* 2009, Goecks *et al.* 2010, Cock *et al.* 2015). Furthermore, Galaxy provides a REST API that can be used to execute computational jobs programmatically and remotely. This allows the use of a Galaxy server as a centralized endpoint for BLAST+ and DIAMOND searches and besides this, any Galaxy server can also be used as a reusable and centralized repository for BLAST+ and DIAMOND sequence databases. However, the generic and tool-agnostic design of the Galaxy REST API makes it difficult to use the NCBI BLAST+ tools or DIAMOND via the Galaxy API conveniently and in an easy manner. For example, parameter names of the Galaxy-wrapped tools when using the Galaxy API are not documented and have to be inferred from the source code of the tool definitions or from HTTP requests when using the tools in the GUI of Galaxy.

So far, a high-level Python API for BLAST+ and DIAMOND searches against Galaxy servers is not available. This motivated us to develop a reusable high-level API layer on top of the Galaxy REST API to simplify the daily use of Galaxy servers for executing BLAST+ and DIAMOND search queries from Python applications or from the command line.

## 2 Implementation and features

**Figure 1.**
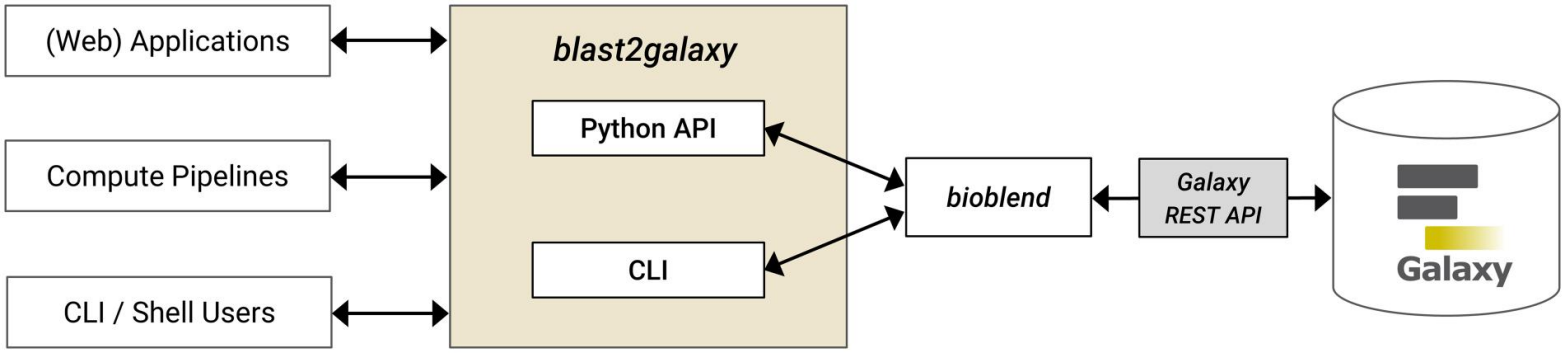
Blast2galaxy provides a high-level convenience layer between any Galaxy server with NCBI BLAST+ tools and/or DIAMOND installed and different types of clients and usage scenarios. Researchers, applications, and computational pipelines can either use the Python-API or the CLI of blast2galaxy to send requests for a BLAST or DIAMOND search to any compatible Galaxy server. The use of BLAST+ tools and/or DIAMOND by multiple applications and the provision of corresponding BLAST and DIAMOND databases can be centralized and made reusable by use of a Galaxy server.

Blast2galaxy is implemented in Python and uses the bioblend package for high-level access to the Galaxy REST API (Sloggett *et al.* 2013). It can be used in two ways: (i) as CLI where blast2galaxy mimics the CLI parameters and options of the NCBI BLAST+ tools and DIAMOND, and (ii) as Python API which allows to integrate BLAST+ or DIAMOND searches via Galaxy servers conveniently in any Python-based application, e.g. a web service or scripts of bioinformatics pipelines. The usage possibilities and the general architecture are illustrated in Fig. 1. To connect to the REST API of a Galaxy server, a user of blast2galaxy has to provide access credentials in the form of an API key which can be obtained on the user settings page of a Galaxy user account.

In addition to the text, HTML and XML result formats known from the BLAST+ tools and DIAMOND, blast2galaxy also allows to format the results as a list of Python dictionaries which considerably simplifies the conversion of the results to a JSON data structure that can be easily consumed by the client-side source code of Javascript-based web applications. DIAMOND can provide the alignment results in JSON format natively as it is a built-in feature. Blast2galaxy can be configured in two ways: (i) using a configuration file in TOML format located either in the user’s home directory or in the current working directory and (ii) using the Python API (Preston-Werner 2013). While the TOML file-based configuration can be used for both CLI and Python API use, the configuration via Python API can only be used when using the Python API. The configuration via the Python API offers the flexibility to configure blast2galaxy from another configuration settings source, e.g. environment variables of a shell or a customized database for configuration settings. The configuration file in TOML format allows to configure multiple Galaxy servers with different server URLs and API keys and also multiple profiles for different BLAST+ and DIAMOND tools (blastn, blastp, diamond blastx etc.). Please refer to the software documentation available at https://blast2galaxy.readthedocs.io for detailed information about configuration options. To ensure functionality and quality of the software tool several end-to-end-tests for the CLI and API interface have been developed, that test blast2galaxy in a real world scenario against publicly available Galaxy instances. The tests conduct multiple BLAST+ and DIAMOND searches for well-known genes and check if the gene can be found in the search results.

## 3 Use cases

One use case for blast2galaxy is the utilization in scientific web services like genome databases and genomic data portals that use Python as the server-side programming language. In this case, the Python API of blast2galaxy can be used to set up a customized domain-specific BLAST search service that sends the search requests to a compatible Galaxy server. Web applications written in another language than Python could use the CLI of blast2galaxy via system command calls and the usage of temporary files for storage of the query sequence.

As sequence searches using BLAST and DIAMOND are a common and regular task in life sciences and especially in the field of genomics and proteomics, a computational biologist or bioinformatician can avoid the necessary and time-consuming creation of BLAST databases for often used reference genomes or protein sequence databases by using already available sequence databases for BLAST+ and DIAMOND on any available Galaxy servers. This means that blast2galaxy can be used as a drop-in replacement in scripts and pipelines that use the BLAST+ tools or DIAMOND.

On PanBARLEX (https://panbarlex.ipk-gatersleben.de) blast2galaxy is embedded under the “Homology Search” feature and used to perform BLAST+ and DIAMOND searches against 3.8 million protein sequences of the barley pan-genome. The alignment results are enriched with information about orthogroup membership of the genes found by BLAST or DIAMOND hits by looking up each hit in the data warehouse of PanBARLEX on the server-side application code. With this feature, users can directly see whether the genes found with their query sequence are located within a single orthogroup or are spread over multiple orthogroups.

In a DivBrowse instance for genomic variants of the barley pan-genome (https://divbrowse.ipk-gatersleben.de/barley_pangenome_v2/), blast2galaxy is used to perform BLAST+ searches against the Morex V3 reference genome of barley (Mascher *et al.* 2021, König *et al.* 2023). The start and end coordinates of the BLAST+ hits are then used in a second step to count the number of genomic variants located within the bounds of each BLAST+ hit. The BLAST+ hits and the corresponding number of variants for each hit are then displayed to the user as a table in the GUI of DivBrowse. The Python API of blast2galaxy is integrated into the Python-based source code of DivBrowse. This feature allows users to find genomic variants like single nucleotide polymorphisms (SNPs) or insertions and deletions (INDELs) for their DNA or amino acid sequence of interest. Here, the BLAST results are enriched with the number of SNPs and INDELs located within the genomic interval of the alignment of the BLAST hits.

## 4 Conclusion

While the direct use of the Galaxy REST API for BLAST and DIAMOND searches is relatively inconvenient due to the generic and abstract design of the Galaxy REST API, blast2galaxy offers a high-level convenience layer for usage in Python applications and on the command line. With blast2galaxy BLAST or DIAMOND searches against Galaxy servers can be conducted using a single CLI command or a single method call of the Python API. That makes the use of a Galaxy server for sequence searches much easier. The integration of BLAST and DIAMOND searches with existing Galaxy servers reduces deployment effort and server maintenance costs for generic and species-specific sequence searches and offers computational resources that might not be available to the user otherwise. The Python API of blast2galaxy allows easy integration of BLAST and DIAMOND searches with Python-based web application backends that makes the development of species-specific and highly integrated sequence searches with customized graphical user interfaces faster and less complex. For Galaxy server operators, it is also possible to use their already existing Galaxy instances with blast2galaxy for BLAST and DIAMOND searches that do not require or cannot support the Galaxy graphical user interface.

## Data Availability

blast2galaxy is available as open-source software under the MIT license. The source code is available on Github: https://github.com/IPK-BIT/blast2galaxy. It can be installed from the Python Package Index using “pip install blast2galaxy” or from the Bioconda channel using “conda install -c bioconda blast2galaxy”. Docker and Apptainer images are available and referenced in the documentation which is available under https://blast2galaxy.readthedocs.io.
